# Research Progress on Vegetable Oil-Based UV-Curing Resins

**DOI:** 10.3390/polym17141890

**Published:** 2025-07-08

**Authors:** Wei Wang, Zhengru Hu, Wen Lei

**Affiliations:** College of Science, Nanjing Forestry University, Nanjing 210037, China

**Keywords:** biobased, vegetable oil, photopolymerization, resin

## Abstract

As a large class of natural organic compounds, vegetable oil is generally composed of 95% fatty acid triglycerides and very few complex non-triglycerides. It has many advantages, such as sufficient yield, low price, distinct structural characteristics, and biodegradability. UV curing technology is known as a new method for the green industry in the 21st century due to its high efficiency, economy, energy conservation, high adaptability, and environmental friendliness. Therefore, UV-curable resins based on UV-curing technology has attracted widespread attention, converting epoxy soybean oil, castor oil, tung oil and other vegetable oils into high-performance plant oil-based UV-curable resins with higher molecular weight, multi-rigid ring and high reactivity, and the curing performance has been greatly improved, and the technology has been widely used in the field of polymer materials such as coatings, inks and adhesives. In this article, the recent research progress on this topic was summarized, and emphasis was put on the research on the resins from soybean oil and castor oil.

## 1. Introduction

At present, the global stone resources are over-consumed. Petroleum derivatives have an impact on the environment and human health. There is a growing consumer demand for environmentally friendly materials. The development of green, environmentally friendly, and high-performance products has become a new development trend [1]. Vegetable oil-based polymer is a promising green polymer. Its raw material sources, production processes, and product applications are all sustainable and environmentally friendly. Many vegetable oil derivatives have been developed from vegetable oils. It is widely used in plasticizers, biodiesel, lubricating oils, adhesives, biodegradable packaging materials, printing inks, coatings, and other fields. Therefore, it is necessary to use vegetable oils to create new ecologically and economically viable polymer materials [2,3].

## 2. Research Background of Vegetable Oil-Based UV-Curable Materials

**Figure 1:**
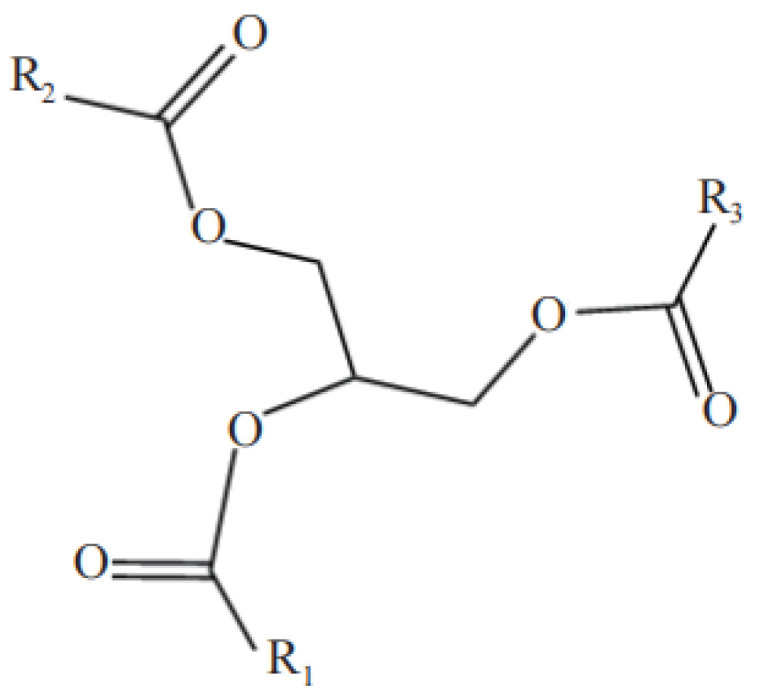
General molecular structural formula of vegetable oils.

Vegetable oils have a unique and versatile component: triglycerides of unsaturated fatty acids, the composition of which depends on the type of fatty acid and the climatic conditions under which it is grown, as shown in Figure 1 [4].

Some fatty acids contain highly reactive groups, such as castor oil, containing hydroxyl groups, and tung oil and linseed oil, containing active conjugated double bond systems. Vegetable oil does not contain end double bonds, and the large steric hindrance leads to low double bond polymerization activity; as a result, it is difficult to be directly applied to the polymer field by polymerization. These particular components offer various possibilities for their modification, of which epoxidation is probably the most well-known and commercially significant. Epoxy vegetable oils can be further chemically converted into acrylic acid or methacrylic acid compounds, i.e., bio-based epoxy acrylate resins, by ring-opening of ethylene oxide rings [5]. Vegetable oil epoxy acrylates containing acrylate groups are widely used in ultraviolet (UV) curing technology.

UV curing is a photochemical process that transforms simple materials into complex structures after exposure to UV radiation [6]. This technology does not evaporate the solvent during the curing process, and it requires about 60 J less energy than the heat energy used in conventional thermal polymerization and forms a dry and fully cured material in a matter of seconds [7]. Today, UV radiation stands out as a widely used curing technology because of its “5E concept”: high efficiency, energy saving, enablement, economy, and environmental protection. Therefore, the combination of UV curing technology and bio-based materials provides a “double green” solution for industry [8,9,10,11]. UV-curable materials are typically formed by light-curing reactions of reactive diluents and oligomers, photo initiators, and additives with unsaturated double bonds. Based on its structural characteristics, vegetable oil has good application potential in the field of light-curing materials. Firstly, the double bonds on the molecular structure of vegetable oil can theoretically be directly cured by ultraviolet light or converted into epoxy groups for curing. Secondly, the active groups, such as double bonds, hydroxyl groups, and ester groups in the structure of vegetable oil, can be converted into highly active light-curing monomers or prepolymers through chemical modification.

Modified and functionalized vegetable oils have become a hot topic for the development of sustainable functional materials, including but not limited to polymers [12], coatings [13], composites [14], and nanostructures [15]. The significant growth of these materials and their continuous optimization at the academic level has led to their use in industry. Previously, the frontier of research was soybean oil, castor oil, tung oil, and flax oil, which are typical representatives of unconjugated highly unsaturated vegetable oils, highly unsaturated dry vegetable oils with conjugated double bonds, hydroxyl vegetable oils, and low unsaturated vegetable oils with conjugated double bonds (Table 1), and some of them have become commercial products, such as epoxidized soybean oil (ESO) and epoxidized sucrose soybean oil (ESS) [16].

This review is finished based on the literature mainly published between 2018 and 2025 (accounting for >80%), emphasizing the research progress in the past five years. The raw materials are diverse, covering major vegetable oils such as soybean oil, castor oil, flaxseed oil, tung oil, and cottonseed oil. The cutting-edge technologies involved include click chemistry, 3D printing, self-healing functions, etc. Applications include coatings, adhesives, 3D printing, composites, etc.

## 3. Research on Vegetable Oil-Based Ultraviolet Curing Materials

### 3.1. Soybean Oil

Soybean oil is one of the most common renewable green resources in human society, and many studies have shown that it can be widely used in many industries, as a highly unsaturated vegetable oil containing non-conjugated bonds [17], its main fatty acid composition includes 50~55% linoleic acid, 22~25% oleic acid, 10~12% palmitic acid, and 7~9% linolenic acid. The soybean oil is low reactive by itself and cannot be directly used as a raw material for ultraviolet photoreaction polymerization, but due to its unsaturated double bonds and ester bonds, it can be structurally modified to prepare monomers or resins with higher reactivity, and the main methods are epoxidation ring-opening modification [18,19,20,21], mercapto-ene modification, and alcohol hydrolysis modification [22].

#### 3.1.1. Epoxidation Ring-Opening Modification

In the resin industry, soybean oil and oxidants can be quickly synthesized into epoxidized soybean oil (ESO) by a simple process with high yields. Epoxidized soybean oil is of great significance in the field of resin synthesis due to its non-volatility, excellent thermal and photostability, and good miscibility with resin materials [23].

**Figure 2:**
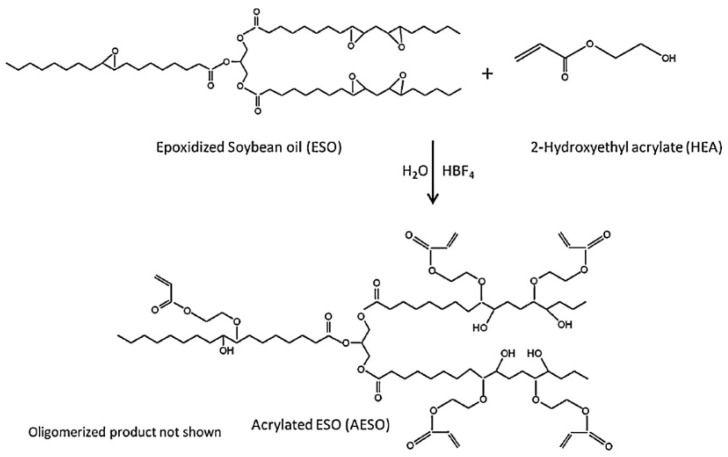
Synthesis protocol for acrylatylated ESO in the presence of a strong acid catalyst.

Rengasamy et al. [24] synthesized monomers and polymers with low viscosity and high content of acrylate using hydroxyethyl acrylate (HEA) and ESO through a superacid-catalyzed etherification reaction, as shown in Figure 2.

**Figure 3:**
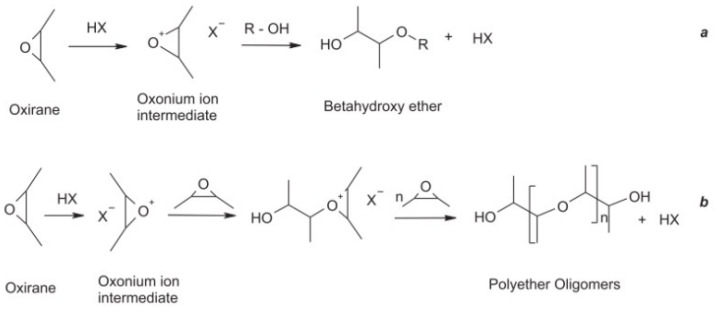
In the presence of strong acids, ethylene oxide compounds compete with alcohols to form (**a**) β-hydroxyether and (**b**) polyether oligomers.

In the presence of strong acids, ethylene oxide compounds competed with alcohols to form β-hydroxyether and polyether oligomers (Figure 3). Hydroxyethyl acrylate (HEMA) was used to replace the acrylic acid in the traditional method to produce an acrylic ether, and then AESO with low viscosity was synthesized, and the results showed that these AESO oligomers had good application prospects in renewable UV-curable coatings.

**Figure 4:**
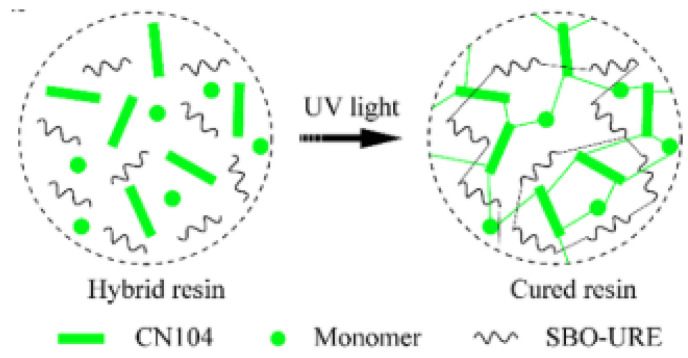
Schematic diagram of the dual-curing system.

Tert-butyl acetoacetate is an important class of general-purpose synthetic intermediates for thermosetting resins, and its functional groups can be efficiently crosslinked with various compounds, such as isocyanates, acrylates (Michael reaction), amines (enamine formation), and aldehydes. Cui et al. [25] described a modified soybean oil with an epoxy group (carbamate epoxidized soybean oil SBO-URE) to enhance the impact strength of aliphatic cyclic diepoxides through a photo-initiated cationic reaction. A dual-cure hybrid resin combining this bio-based resin with acrylates was reported, which could be used to prepare high-performance thermoset SLA-3D printing materials. In this dual-cure system, a mixture of acrylates (with radical photo initiators) and epoxidized vegetable oils (with cationic photo initiators) could be polymerized simultaneously under exposure, as shown in Figure 4. The results showed that the introduction of photo-initiated cationic SBO-URE ensured that the printed matter had sufficient strength to form an interpenetrating network structure, without significantly reducing the elongation due to the presence of long fat chains.

**Figure 5:**
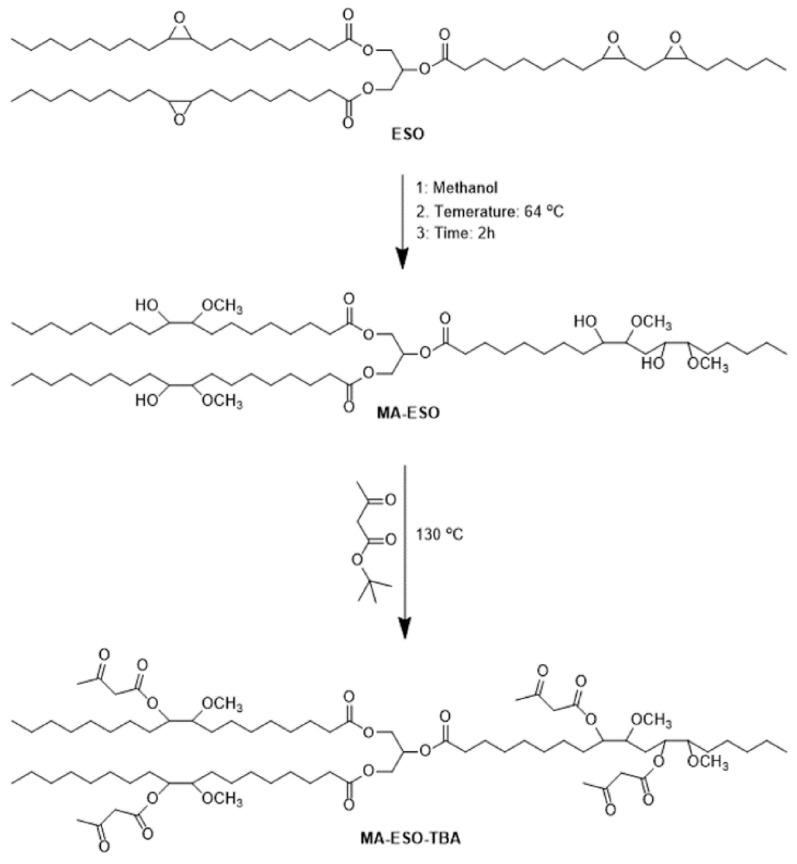
Acetylacetylated soybean oil is synthesized by ring-opening and transesterification.

Cao et al. [26] took advantage of this property to prepare acetoacetylated soybean oil by ring-opening and transesterification reactions (Figure 5), where soybean oil-based polyols were first synthesized by opening the cycles in the soybean oil with methanol, and then acetoacetylated soybean oil was synthesized by transesterification with tert-butyl acetoacetate. Finally, acetoacetylated soybean oil was used as a raw material to prepare several bio-based coatings with different aromatic dialdehydes; the Young’s modulus, tensile strength, and elongation at break of the coatings could be as great as 24.91 MPa, 5.65 MPa, and 286%, respectively. Zhang et al. [27] synthesized soybean oil-based biopolyols by ring-opening reaction of epoxy soybean oil (ESO) using glycerol as a nucleophile and ionic liquid as a green and efficient catalyst, and the impacts of various reaction variables on the ring-opening reaction were systematically investigated employing one variable at a time (OVAT) and response surface methodology (RSM). They underscore the potential of sulfonic-functionalized [BSO3HMIM]OTF IL as an efficient and eco-friendly catalyst in sustainable biopolyol production.

#### 3.1.2. Mercapto-Enes Modification

One of the most attractive “click” reactions in polymer chemistry is the thiol-alkene-coupling (TEC) addition reaction, which is mainly used for the crosslinking of fatty acids and triglycerides through the use of polyfunctional thiols [28]. In addition, the lubricating properties of vegetable oils are effectively optimized by TEC reaction or after grafting mercaptosilanes on metal surfaces. The thiol-alkene coupling reaction has the advantages of being simple, rapid, and high in yield [29], and can also be used to increase the hydroxyl content in polymers, it has been gaining great attention due to its ease of implementation and high efficiency, and this method has actually been widely used for polymer networks, custom macromolecules, bioconjugated synthesis, chemical modification of surface and nanostructures, and post-functionalization. In this context, many studies have been conducted on the free radical addition of multifunctional mercaptans to vegetable oils [30].

**Figure 6:**
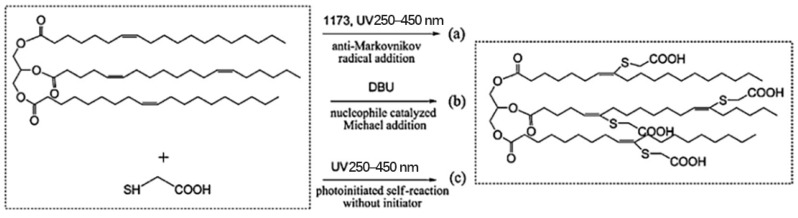
Route of thiol-olene addition reaction. (**a**) anti-Markovnikov radical addition (**b**) base or nucleophile catalyzed Michael addition reaction (**c**) photoinitiated thiol-ene self-reaction without initiator.

**Figure 7:**
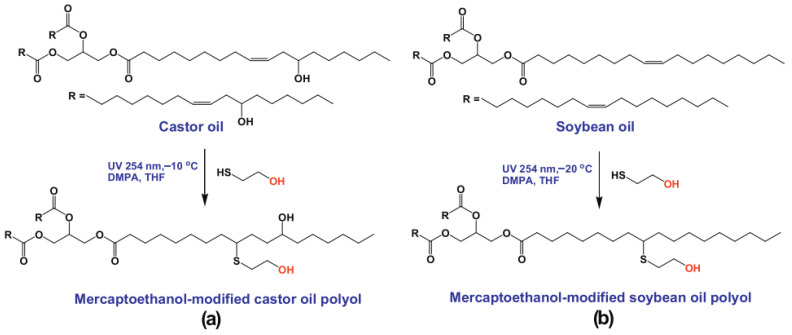
Polyols with primary hydroxyl groups were prepared by the thiol-ene reaction of castor oil (**a**) and soybean oil (**b**) with ME.

Desroches et al. [31] performed a model study of oleic acid and 2-mercaptoethanol; the experimental parameters of the mercaptoethanol and oleic acid addition reaction were optimized and then applied to the functionalization of soybean oil. It was found that the number of double bonds per chain in vegetable oils affected the thiol grafting rate, that reaction time and by-products could be reduced by optimizing experimental conditions, and that photoreactions could be carried out under mild conditions without the need for either solvents or photo initiators, and that crude products could be purified in simple steps. He et al. [32] synthesized UV-cured soybean oil-based multifunctional acrylates (PFAs) by photo click thiol-ene reaction and DCC (N, N-dicyclohexylcarbodiimide) -catalyzed esterification reaction, and different functional groups were introduced into fatty acids (esters) by the thiol-alkene addition reaction, and intermediates were formed, providing a new pathway for the rapid and efficient synthesis of soybean oil-based acrylates, as shown in Figure 6. The structure of soybean oil-based acrylates was characterized by 1H NMR and FTIR, and the results showed that almost 100% conversion of double bonds in vegetable oil was observed within 16.7 min, and soybean oil-based polycarboxylic acids were quantitatively produced. Moreover, PFA was observed to have an excellent UV curing rate, which could be used to prepare high-performance UV curing materials. Alagi et al. [33] optimized the thiol-alkene coupling reaction of castor oil and soybean oil-based polyols with a C-C bond conversion of more than 99%, as shown in Figure 7. By adjusting experimental parameters such as the concentration of thiol compounds and the reaction temperature, the reaction efficiency was improved, and for the first time, the C-C double bond of vegetable oil was almost completely and quantitatively converted to hydroxyl groups, and their research succeeded in introducing polyols into TPU to obtain elastomers with an overbranched chain structure.

**Figure 8:**
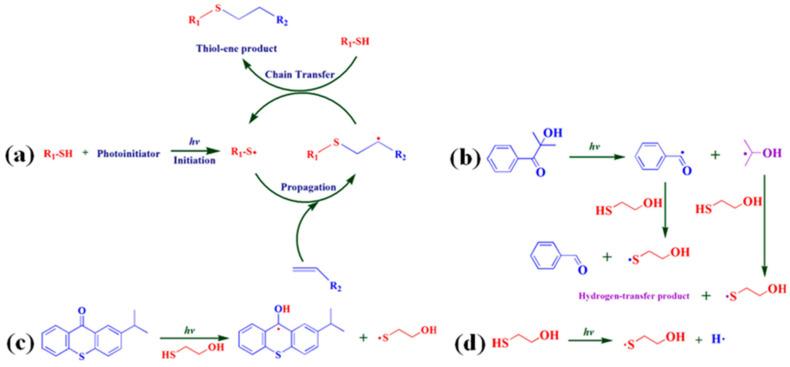
Mechanism of thiol-ene photoclick reaction (**a**) and sulfur-based radical formation initiated with 1173 (**b**), ITX (**c**), and no photoinitiator (**d**).

Feng et al. [34] developed a solvent-free method for the large-scale preparation of soybean oil-based polyols by the thiol-ene photo click reaction, and the mechanism of the reaction was discussed (Figure 8). Several polyurethanes were prepared using different diisocyanates (aliphatic, alicyclic, and aromatic isocyanates), and the characterization results showed that the resulting polyurethane films had good properties, with a maximum glass transition temperature of 41.3 °C, a tensile strength of 15.7 MPa, and an elongation at break of 471.0%.

#### 3.1.3. Alcohol Hydrolysis Modification

Alkyd resin (AR) is defined as a polyester with unsaturated fatty acids, and its raw materials are mostly bio-based, making AR an “eco-friendly” polymer commonly used as a binder in coatings or paints. These resins have excellent gloss, heat resistance, and other characteristics, and their properties can be adjusted by changing the type of oil (soybean oil, flaxseed oil, tung oil). However, AR has poor wear resistance, UV resistance, and chemical resistance, which limit its wide application. Therefore, the focus of industrial and academic research is to improve the properties of alkyd resins through additives or polymers [35].

**Figure 9:**
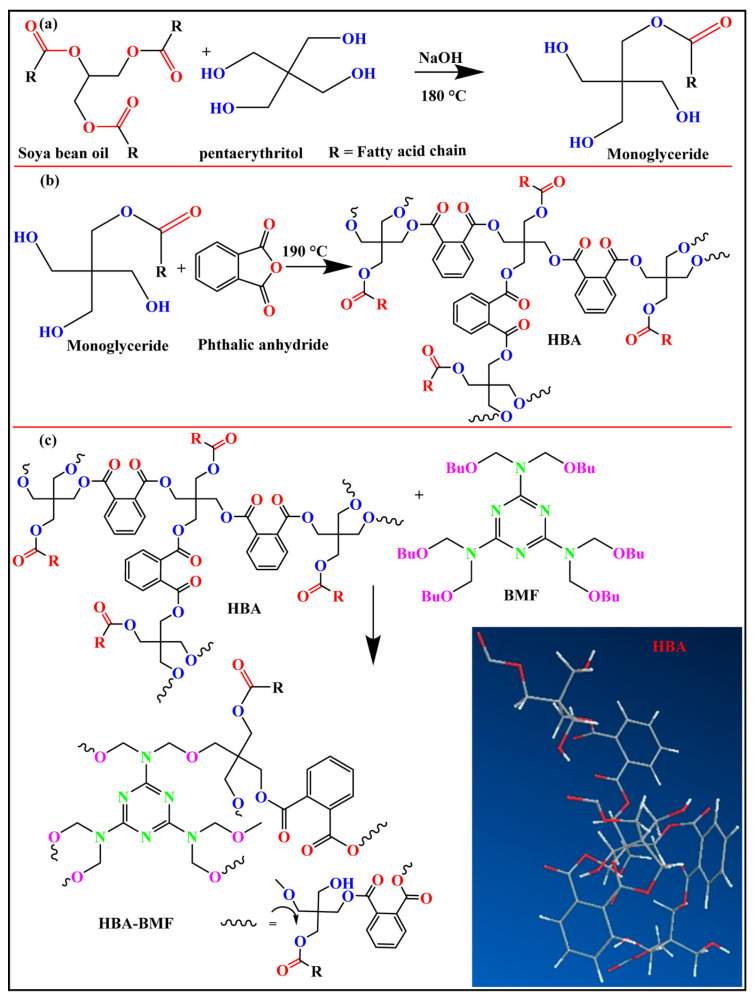
Synthesis of (**a**) monoglyceride, (**b**) hyperbranched alkyd (HBA), (**c**) HBA−BMF, and in inset 3D view of hyperbranched HBA.

Rahman et al. [36] reported the synthesis and corrosion inhibition performance of hyperbranched soy alcohol acid nanocomposite coatings. They synthesized hyperbranched alcohol acid resin (HBA) using soybean oil, pentaerythritol, and phthalic anhydride as raw materials, and the reaction principle is shown in Figure 9. Nano Fe_3_O_4_ was dispersed in butylated melamine formaldehyde (BMF) modified HBA (HBA-BMF) using the ultrasonic dispersion method to prepare a nanocomposite (HBA-BMF-Fe_3_O_4_) anti-corrosion coating. The structure, morphology, physical and mechanical properties, thermal properties, electrochemical properties, and corrosion resistance of these coatings were evaluated using the ASTM method, and the results showed that this nanocomposite coating had excellent corrosion resistance and other properties.

**Figure 10:**
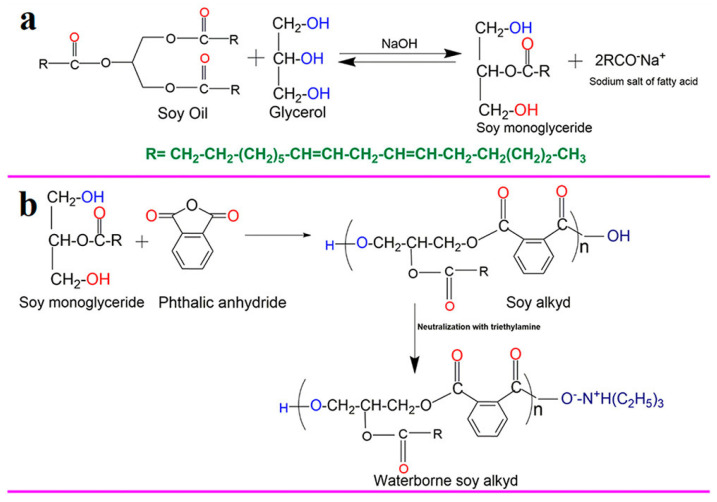
Reaction scheme for the synthesis of (**a**) SMG, (**b**) WSA.

Irfan et al. [37] synthesized waterborne alkyd resin (WAR) using soybean oil monoglyceride (SMG) as a precursor through a non-in situ polymerization method. The reaction principle was shown in Figure 10. The synthesis of SMG was realized by reacting soybean oil, glycerol, and NaOH in a certain ratio, which was an alkaline catalytic reaction. The synthesis of waterborne soy alcohol acid resin involved mixing SMG and phthalic anhydride at 190 °C and heating under constant stirring for 3.5 h. Then, triethylamine and ethanol solution were added to the reactants in a ratio of 70:30 to form the free terminal carboxyl groups of waterborne soy alcohol acid resin. In addition, they also modified WAR with nano fillers and melamine formaldehyde isobutyric acid solution and studied its synergistic effect on the physical and mechanical properties and corrosion inhibition of coatings on polished carbon steel. The results indicated that this nanocomposite coating had good protective ability, high mechanical stability, and environmental friendliness, making it a potential commercial application prospect in the field of anti-corrosion coatings. Chukwuebuka et al. [38] studied the process conditions for producing alkyd resin from dehydrated castor oil and soybean oil as raw materials. The standardized results of coating analysis on soybean and castor oil modified resins showed that they both had good commercial properties in terms of chemical resistance, adhesion, solubility in solvent media, and drying time. However, under the same conditions, soybean oil-modified resin had better quality due to its lower acid value.

### 3.2. Castor Oil

Castor oil (CO), one of the most promising vegetable oils, is widely used in the design of UV-curable polyurethane materials, mainly due to its natural hydroxyl group [39]. These groups can be polycondensed with isocyanates to form hard segments that give excellent mechanical strength to the polymer, while long-chain fatty acids on CO can give the structure some flexibility. Castor oil is a large-scale, industrially produced biomass raw material containing functional groups such as hydroxyl groups, double bonds, and ester groups, which can be used as a raw material for the synthesis of polyurethanes. However, the low hydroxyl content in castor oil usually makes the prepared polyurethane haver poor performance, consequently, the material can hardly be utilized directly, meanwhile, the polyurethane products need to face external damage, scratching, and chemical corrosion when in use, all these will worsen the material properties and their service lives will be seriously affected, so it is necessary to carry out modification on CO [40]. The most common modification methods are to structurally modify the native reaction sites in triglycerides and unsaturated double bonds [41] by introducing more hydroxyl groups. Increasing the hydroxyl value and functionality of castor oil molecules can significantly improve their reactivity, making them more suitable as a polyol raw material for the synthesis of high-performance polymers, especially bio-based polyurethane materials. The increased hydroxyl group provides more reaction sites and enhances crosslinking with monomers such as isocyanates, ultimately improving the mechanical properties (e.g., strength, toughness), thermal, and chemical stability of the polymer.

#### 3.2.1. Mercapto-Ene Modification

**Figure 11:**
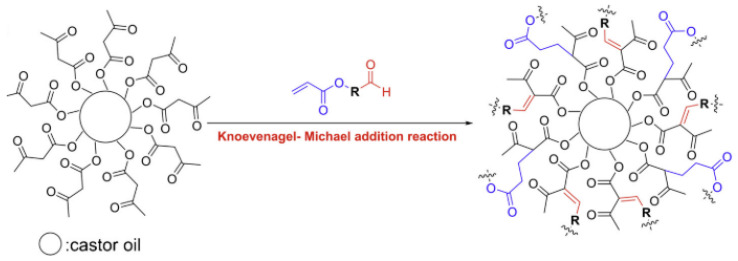
Knoevenagel and Michael addition reactions.

**Figure 12:**
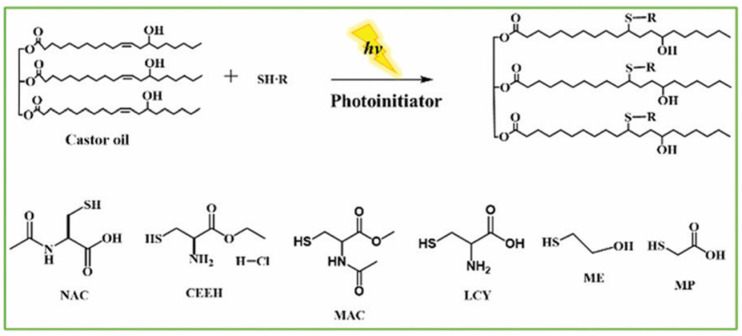
Internal emulsifier prepared with different mercaptans and castor oil.

Xu et al. [42] prepared modified acetoacetylated castor oil by thiol-alkene coupling and transesterification, and then a novel environmentally curing coating film was prepared through Knoevenagel and Michael addition reactions between the modified acetoacetylcastor oil and a crosslinker, which contained aldehyde and acrylate groups, as shown in Figure 11. The properties of the coatings prepared by Knoevenagel addition reaction, Michael addition reaction, and the combination of Knoevenagel and Michael addition reactions were compared, and the results showed that the coatings obtained from the combination of Knoevenagel and Michael addition reactions had a good balance of properties. Wang et al. [43] used natural castor oil as a raw material to perform a photo click thiol-ene reaction (Figure 12) for the synthesis of bio-based internal emulsifiers, with a conversion rate of up to 92.0%. This inner emulsifier was further applied to the synthesis of waterborne polyurethane polyols with excellent physical and mechanical properties and a bio-content of up to 90.0 wt%. Natural materials, such as amino acid derivatives and castor oil, could be combined with petroleum-based products via this novel emulsifier chemically prepared by photoclick thiol-ene, providing a green and sustainable strategy for the synthesis of waterborne polyurethanes. Liang [44] synthesized mercaptocastor oil (MCO) by mercapto-olefin reaction using castor oil and mercaptoacetic acid as raw materials, and esterified it with glycidyl methacrylate (GMA), and a novel trifunctional castor oil-based methacrylate oligomer (MCOG) was accordingly synthesized. Using MCOG as the main ingredient and compounding it with the active monomer pentaerythritol triacrylate (PETA), photo initiator, and another self-made polyurethane acrylate oligomer (B-215), a light-curing coating with certain properties was prepared. Su et al. [45] provided a green and efficient method for the synthesis of vegetable oil-based polyols with ultra-high hydroxyl value to construct high-performance polyurethanes. The 1-thioglycerol-modified castor oil with an ultra-high hydroxyl value of up to 463 mg KOH g−1 was synthesized by solvent-free mercapto-olefin click reaction using castor oil. The polyol had been used successfully to prepare cross-linked polyurethanes without solvent and catalyst; the tensile strength of the obtained products was much higher than that of other reported vegetable oil-based polyurethanes. In addition, the prepared polyurethane exhibited excellent coating and optical properties, which were far superior to those of the polyurethanes from unmodified castor oil.

#### 3.2.2. Polyurethane Acrylic Modification

Acrylate-based vegetable oils represent one of the most abundant renewable raw materials in UV-curable materials and have many applications in paints, varnishes, and related materials. There are several types of functionalization, such as the reaction between (meth)acrylic acid and epoxidized oil [46], the esterification of (meth)acrylic acid with a vegetable oil with a hydroxyl group [47], and the reaction between the terminal isocyanate group of a vegetable oil and an acrylate monomer with a free OH group [48], which can obtain ester groups, allyl carbon, and epoxy groups at various active sites such as double bonds within the triglyceride structure.

**Figure 13:**
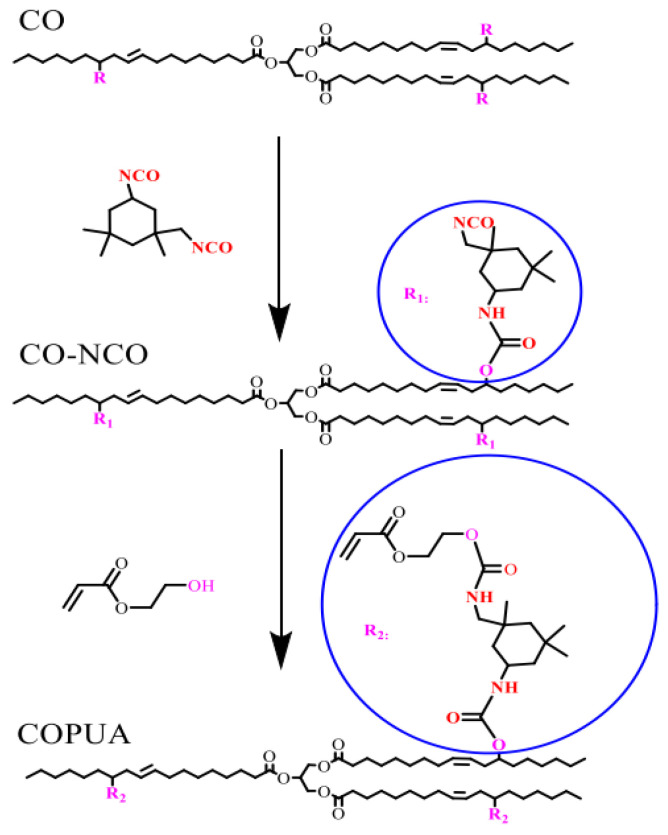
Synthetic route of COPUA.

Hu et al. [49] synthesized a highly functional castor oil-based PUA premer (COPUA) by a two-step method using castor oil, isophorone diisocyanate, and pentaerythritol triacrylate, and the structure of the products was confirmed by FT-IR, ^1^H NMR, and gel chromatography (GPC). The obtained COPUA was blended with the petroleum-based reactive diluent hydroxyethyl methacrylate (HEMA) and then cured by UV to prepare the light-curing material, and the final properties and light-curing kinetics of the obtained materials were investigated. The results showed that the pure COPUA resin material had excellent properties. Li et al. [50] synthesized COPUA using CO, isophorone diisocyanate (IPDI), hydroxyethyl acrylate (HEA), and isobutyl acrylate (IBOA) as raw materials, as shown in Figure 13. The curable UV films were prepared by mixing COPUA, photo initiator (PI-1173), monofunctional reactive diluent hydroxyethyl methacrylate (HEMA) or diluent tripropylene glycol diacrylate (TPGDA), and the results showed that the cured films had excellent thermal stability, storage modulus (421 MPa at 25 °C) and tensile strength (9.87 MPa) as well as high glass transition temperature (60.3 °C). In this study, the viscosity of the reaction system was reduced, and almost 100% raw materials were utilized, saving time and energy, and the solvent could be UV cured, which improved the curing performance.

Cheng et al. [51] developed a highly transparent silicone-modified polyurethane acrylate UV-curable coating with excellent tensile strength and chemical resistance using castor oil-based polyurethane acrylate and sulfhydryl silicone resin as substrates. The UV-curable coating had a transparency of 90–98%, a tensile strength of up to 10.2 MPa, and an elongation at break of 88.4–162.5%. After immersion in 3.5 wt% hydrochloric acid aqueous solution for 144 h, the tensile strength of the coating was increased from 6.2 MPa to 15.5 MPa, while the elongation at break hardly changed, indicating that the UV-cured coating had good chemical resistance properties. Zhu et al. [52] designed bio-based photopolymers for recyclable DLP-printed packaging by hindered urea bonds (HUBs). A novel CO-based polyurethane oligomer (COIT) was first synthesized and then blended with 2-(tert-butylamino)ethyl methacrylate (TBEM) to prepare DLP printing resins. By heating the material with additional TBEM, the printed object could be easily depolymerized into a liquid resin, and a reprintable resin could be obtained by adding some new COIT oligomers to the depolymerized resin. In the absence of any catalyst or solvent, the printed material was completely depolymerized at 90 °C for 4 h or 100 °C for 2 h. At the same time, the physicochemical properties of the recycled resin, such as viscosity, tensile strength, and glass transition temperature (T_g_), were almost identical to those of the original resin.

### 3.3. Other Vegetable Oils

**Figure 14:**
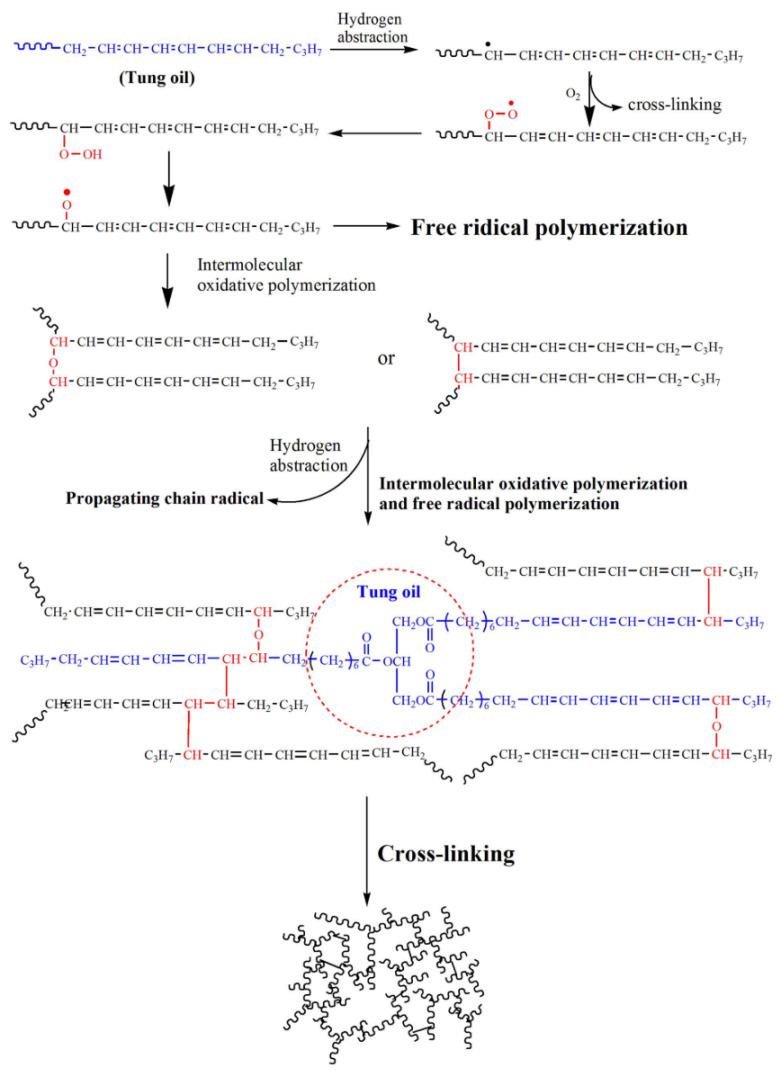
Ultraviolet curing mechanism of tung oil.

Huang et al. [53] studied the curing mechanism of tung oil under ultraviolet light irradiation (Figure 14) and the effects of different photo initiators on the UV curing behavior of tung oil. A novel UV curing system for tung oil was constructed, which provided a reference for further expanding the application of dry oil.

**Figure 15:**
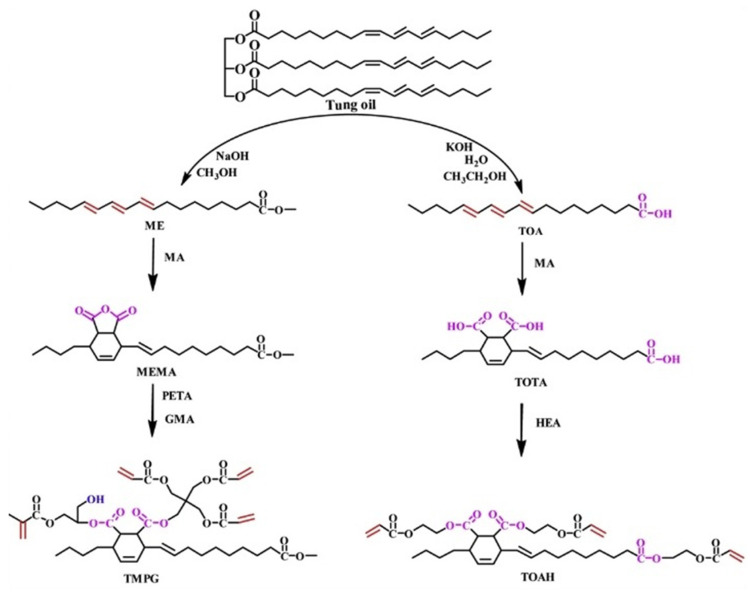
Synthetic routes of TOAH and TMPG.

As illustrated in Figure 15, Liang et al. [54] synthesized two renewable UV-curable TO-based reactive monomers: triacrylate (TOAH) and tetraacrylate tungstate maleate (TMPG) by Diels–Alder reaction and ring-opening esterification reaction. In their study, tung oil-based derivatives (tung oleic acid and methyl tung oleate) were reacted with maleic anhydride, and then the subsequent products were modified with acrylic acid monomers, the obtained monomers had trifunctional and tetrafunctional groups, respectively, and the molecular weight of tung oil in each monomer accounted for more than 35%. When the two monomers were mixed at different mass ratios and cured with a 2,4,4-trimethylbenzoylphenylphosphonate ethyl ester (TPO-L) photo initiator under UV radiation, it was found that the curing properties, dynamic mechanical properties, thermal stability, tensile properties, and chemical resistance of the copolymers were improved.

**Figure 16:**
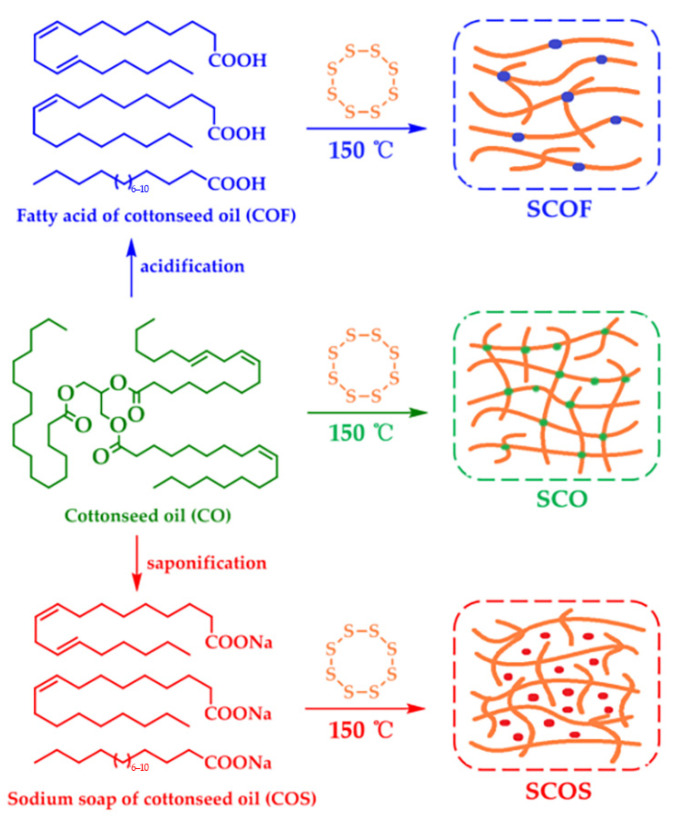
Reaction characteristics of CO, COF, and COS in polysulfide polymers.

Sahoo et al. [55] synthesized environmentally friendly UV-curable oligomers using epoxidation, transesterification, and acrylic esterification and applied them to adhesives. Flaxseed oil was epoxidized and then transesterified to form methyl epoxy ester to reduce viscosity and readily available epoxy groups for a higher degree of acrylate esterification. Epoxy methylacrylate was synthesized using a sodium methoxide catalyst, and acrylic was used for acrylic esterification. Yields, epoxidation, transesterification, and acrylate esterifications had been studied to verify that this environmentally friendly UV-curable oligomer could be used in adhesive applications. Samper et al. [56] developed a thermosetting resin derived from epoxidized linseed oil (ELO) with a high bio-based content by using a hybrid crosslinker of methyl nadic anhydride (MNA) and maleic anhydride linseed oil (MLO). When only MNA was used as the crosslinker, the product obtained from the resin was rigid and brittle, while when MLO was included in the crosslinking mixture by 25 wt%, a reduction in mechanical resistance and thermomechanics was detected (Table 2), thus suggesting that MLO could provide flexibility to thermoset resins, and this research enhanced the potential of this material for use in green composite coatings. Chen et al. [57] designed and successfully prepared polysulfide-derived polymers with controllable density and mechanical strength using bio-based cottonseed oil (CSO) and its derivatives, including cottonseed oil fatty acids (CSOFs) and cottonseed oil sodium soap (CSOS), and the reaction characteristics of CSO, CSOF, and CSOS in polysulfide polymers were comparatively investigated (Figure 16). The results showed that the density and tensile strength of these polymer composites could be affected by the dosage of CSOF, while CSOS did not participate in the reaction with sulfur, it actually acted as a filler, which could increase the density and tensile strength of polysulfide derivatives; meanwhile, the sample had good reworkability and recyclability.

**Figure 17:**
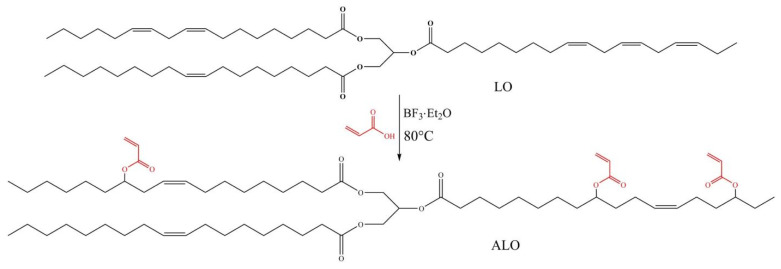
Synthetic route of ALO.

**Figure 18:**
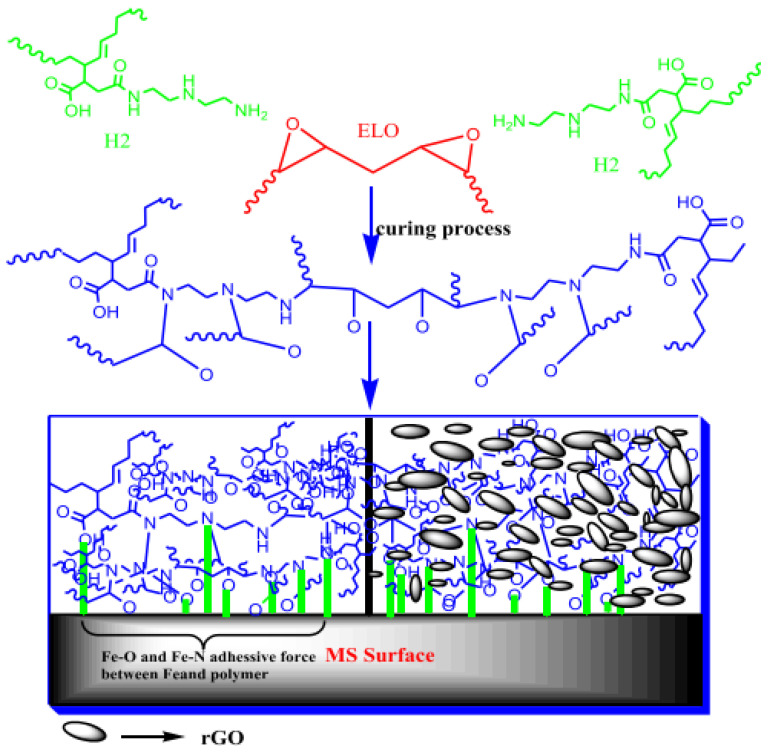
Solidification and corrosion protection mechanisms of coating systems.

With the rapid development of industry and the increasing shortage of fossil energy, tung oil (TO) has attracted extensive attention from researchers because of its environmental protection and renewable advantages. Su et al. [58] proposed a one-step method for the preparation of UV-curable acrylic linseed oil prepolymer ALO using linseed oil (LO) as a raw material (Figure 17). They synthesized a new type of linoleum-based functionalized acrylic acid prepolymer using flax oil and acrylic acid as raw materials and boron ethyl trifluoride ether as catalysts, and prepared a series of ultraviolet curing coatings with trimethylpropane triacrylate (TMPTA), polyurethane acrylate resin, and photo initiator as raw materials. The results showed that as the TMPTA content rose from 0 wt% to 40 wt%, the corresponding tensile strength increased from 6.54 MPa to 13.62 MPa, and the Young’s modulus increased from 64.18 MPa to 361.26 MPa. The glass transition temperature and cross-linking density of the cured film were increased; the glass transition temperature increased from 54.7 °C to 78.9 °C, and the increase in cross-linking density was conducive to the improvement of the thermal stability and mechanical properties of the cured film. At the same time, the cured film exhibited strong adhesion, excellent hardness, and excellent water (solvent) resistance. Hubmann et al. [59] prepared a light-curable film by a combination of epoxidation and transesterification of flaxseed oil, which could be used as an alternative to conventional dry oils. The combination of these epoxidized flaxseed monomers had a shorter cure time; it could cure in just a few minutes. Owing to the formation of flaxseed fatty acid methyl ester, more than 80% epoxy groups were converted into ether crosslinks; as a result, its hydrolytic degradation ability would be reduced by 20%. Hegde et al. [60] focused on the preparation of linseed oil (LO)-based epoxy composite coatings of supported reduced graphene oxide (rGO) on the surface of mild steel. Flax oil was filled with reduced graphene oxide (rGO) and epoxidized to form epoxy resin. Then, the resin was used to coat the surface of mild steel by the spin coating method. The electrochemical properties’ tests showed that the corrosion rate of the composite system on carbon steel was reduced by about 5000 times, the protection efficiency was as great as 99.98%, and the coating could be kept stable in 3.5wt% NaCl solution for 10 days. The curing and corrosion protection mechanism was shown in Figure 18.

**Figure 19:**
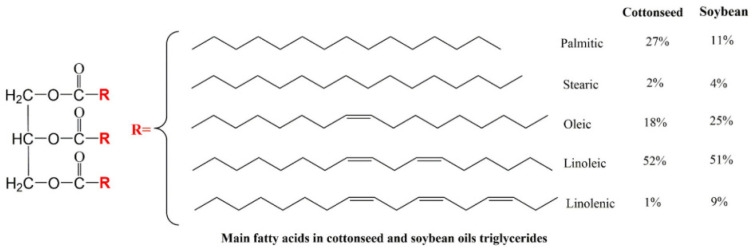
General chemical structure of cottonseed oil and soybean oil.

**Figure 20:**
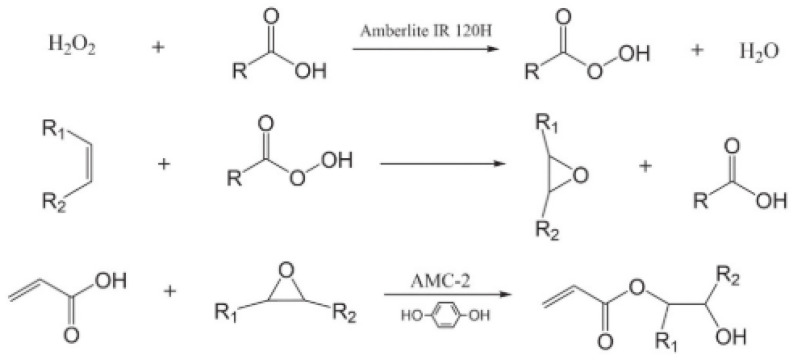
Epoxidation and acrylate reaction protocol for cottonseed oil.

Zhao et al. [61] successfully synthesized a novel bio-based prepolymer (b-acryloyl nutrient ethyl) ester (ARA)/acrylate-2-hydroxyethyl ester (HEA) by reacting tung oil with acrylic modified rosin, and then the prepolymer was used to synthesize a novel bio-based prepolymer-acrylate-epoxy tung oil polymer (AETP). The 3D products printed with AETP had excellent thermal and mechanical properties, with a system viscosity of 313 mPa s, tensile strength, flexural strength, and flexural modulus of 62 MPa, 63.84 MPa, and 916.7 MPa, respectively. Their research pioneered a renewable and low-cost biomass light-curable 3D printing material. Vonsul et al. [62] developed bio-based UV-curable polymer resins using cottonseed oil. Since its fatty acid composition is different from that of soybean oil (Figure 19), cottonseed oil products can be used in different fields. They proposed a combination of cottonseed oil as a raw material and UV curing technology to provide another “greener” solution to the problems faced by industry. Cottonseed oil was first epoxidized using peracetic acid, and then acrylic oxide cottonseed oil (AECO) was synthesized by its reaction with acrylic acid, as shown in Figure 20. A vegetable oil-based film could be prepared by converting AECO into a cross-linking network by photopolymerization. Meanwhile, different weight ratios of 1,6-hexanediol-acrylate and trimethacrylene triacrylate were chosen as the reactive diluents for the products to obtain ideal mechanical properties. It could be concluded from this study that functionalized resins obtained from cottonseed oil could provide a competitive and sustainable alternative for the rapid development of bio-based UV-curable materials.

## 4. Application

**Figure 21:**
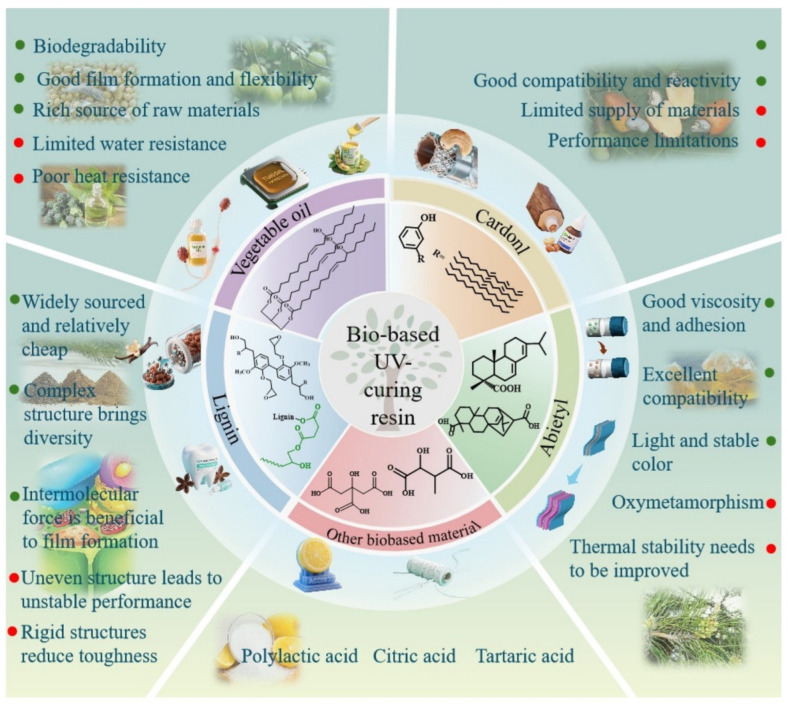
Various applications of bio-based UV-curable resins.

Vegetable oil-based UV-curing resins have been widely used in coatings, adhesives, and 3D printing due to their renewable raw materials, environmental protection, abundant supply, wide distribution, and low price (Figure 21) [63].

### 4.1. Coatings

Paints have a history of thousands of years, and the vegetable oils used in paints in the early days were mainly dry oils. This type of vegetable oil contains more unsaturated double bonds. When they are added to the coating as a binder, cross-linking polymerization will occur when exposed to air, resulting in an increase in molecular weight and molecular chain growth, thereby improving the physical and mechanical properties of the coating film. However, there are defects such as long drying time and poor solvent resistance. After continuous exploration and improvement, researchers have developed a series of vegetable oil-based coatings [64,65,66], which have different characteristics and great market potential.

Yin et al. [67] developed a novel vegetable oil-based UV-curable coating system to synthesize soybean castor oil-based polyurethane acrylate prepolymers using epoxy soybean oil and ricinoleic acid as raw materials, and also synthesized a cost-effective halogen-free flame retardant phosphosilicone methacrylate, which makes these coatings have excellent flame retardant properties. Zhou et al. [68] used dihydroxymethylpropionic acid (DMPA) and methacrylate (MAAH) to modify epoxy soybean oil to prepare high-performance soybean oil-based UV-curable coatings, which significantly improved their thermal stability, tensile, and coating properties. Tuo et al. [69] converted castor oil into a castor oil-based triacrylate structure (MACOG) through chemical modification, and with the increase in MACOG content, the thermal decomposition temperature, mechanical strength, and water resistance of the UV-cured coating were greatly improved, and a waterborne polyurethane coating with excellent comprehensive properties was prepared. The preparation of coatings often requires petroleum-based reactive diluents that are harmful to the environment and human health. Peng et al. [70] developed a novel, environmentally friendly diluent. Their synthetic vinyl-terminated photoactive citric acid derivatives contain many hydroxyl groups that enhance the polarity and cohesive strength of polymer molecules, which can be used to make soybean oil-based UV-curable coatings. Not only does it reduce the use of petroleum-based reactive diluents, but it also improves the performance of soybean oil-based UV-curable coatings. Their group also developed a “green+green+green” method to prepare waterborne epoxy soybean oil-based UV-curable coatings [71]. The epoxy methacrylate obtained by the reaction has excellent hydrophilicity, compatibility, and stability. At the same time, after UV curing, the resulting coating exhibits excellent adhesion as well as thermal stability and mechanical properties. Due to the lack of a rigid structure of traditional UV-curable coatings made of acrylateylated epoxy soybean oil (AESO).

### 4.2. Adhesive

In the field of adhesives, petrochemical-based polymers have been the main choice for a wide range of applications, but relying solely on fossil fuels can lead to problems such as a lack of raw materials and environmental pollution. As a result, the production of polyurethane polymers derived from bio-based feedstocks is of great interest [72,73,74,75]. Bio-based raw materials include renewable resources such as plant-based biomass, agricultural waste, and vegetable oil [76,77,78], among which vegetable oil has become the most promising and most researched raw material in the PU industry due to its abundance, biodegradability, environmental friendliness, and low price [79].

Li et al. [80] synthesized a series of epoxidized soybean oil-based UV-curable polyurethane pigment adhesives based on acrylates, which do not require a lot of water and energy compared to traditional heat-curing coatings, reducing energy consumption and environmental pollution during pigment printing. Yan et al. [81] successfully prepared a series of vegetable oil-based ethanol–water-based adhesives by introducing thymine-containing monomers to increase the adhesive. This adhesive has excellent adhesion properties and can be adjusted by adjusting the UV light intensity. Li et al. [82] used epoxidized soybean oil (ESO) and dithiol borate curing agent (BDB) to prepare a recyclable epoxy adhesive with dynamic borate bonds, which exhibited excellent bond strength on a variety of substrates, including steel, glass, wood, and bamboo.

### 4.3. Three-Dimensional Printing

Most of the resins currently available for UV-assisted additive manufacturing (AM) are made from petroleum-based materials, which is contrary to the concept of environmental friendliness and sustainability. In order to meet societal and industry demands for sustainability, renewable feedstocks must be explored [83,84,85]. Unfortunately, there are not many options for photopolymerization. Nonetheless, some vegetable oils can be modified and made suitable for UV-assisted additive manufacturing by adding reactive diluents [86].

**Figure 22:**
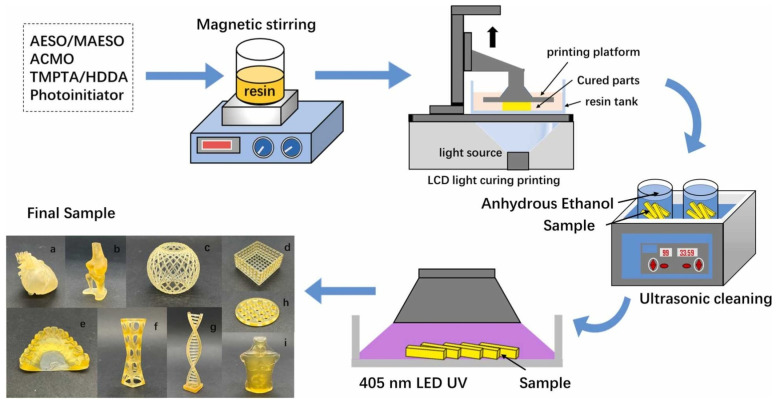
Preparation of soybean oil-based epoxy resin polymer by light-curing 3D printing. (a–i) The model of soybean oil-based epoxy resin was printed by LCD.

**Figure 23:**
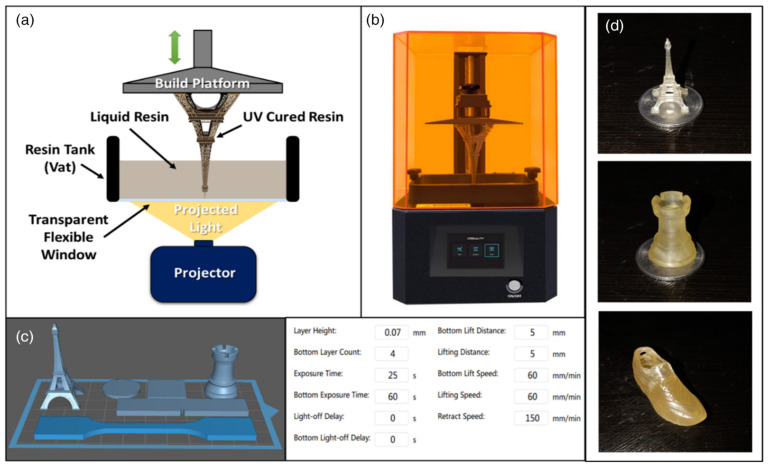
Digital light processing 3D printing (**a**) working principle, (**b**) Creality LD002R LCD resin 3D printer, (**c**) test specimen placement on the build platform and its printing setting, (**d**) 3D printed objects.

Lebedevaite et al. [87] thermally synthesized a calcium silicate hydrate from aluminum fluoride production waste for use as a filler in acrylate epoxidized soybean oil-based light-curing resins, a biodegradable composite material that can be used for 3D printing with high printing accuracy, perfect layer adhesion, and a smooth surface finish. Briede et al. [88] used a syringe-based 3D printer to successfully print photosensitive vegetable oil-based acrylates by extrusion. Adjust and optimize resin properties by optimizing the fatty acid molecular composition of rapeseed, flaxseed, and grapeseed oils. They found that a syringe-based 3D printer approach could print vegetable oil-based resins synthesized in the laboratory and reduce the amount of resin required to a few milliliters, thus solving the problem that commonly used printers require large amounts of resin. Chen et al. [89] developed a vegetable oil-based UV-curable resin that can be used for 3D printing and combined the simulation results with the device parameters to obtain an optimal printing process, which significantly improved the 3D printing efficiency of soybean oil-based epoxy resin, making it have great application potential in biomedical materials (Figure 22). Bhanushali et al. [90] developed a novel UV-curable polyurethane acrylate (PUA) oligomer using castor oil (CO) to prepare a series of UV-curable resins by mixing PUA with different reactive diluents (Figure 23), which can be used for general prototyping applications in the 3D printing industry for the manufacture of architectural models, automotive parts, and medical parts.

The following table (Table 3) is a comparison table of the modification and application of various vegetable oils in the field of ultraviolet curing, which comprehensively considers their raw material characteristics, modification methods, performance advantages, and application scenarios.

## 5. Conclusions

The core of the research of vegetable oil-based UV curing resins is to perfectly combine the sustainability advantages of renewable resources with the high-efficiency and energy-saving advantages of UV curing technology through molecular design and advanced manufacturing technology, and achieve comprehensive performance comparable to or even better than traditional petroleum-based products. In the future, its application will deeply penetrate into a wide range of fields such as coatings, inks, 3D printing, electronics, composite materials, etc., and is one of the key technologies to promote the green and low-carbon transformation of the chemical materials industry. Breaking through the performance bottleneck, reducing costs, and improving standards will be the key to its large-scale industrialization.

Today, plastics are being used in an ever-expanding range of applications, gradually replacing traditional materials such as steel, wood, and glass. UV-curable materials have been used for commercial purposes for more than 30 years, and in recent years, significant progress has been made in the research of vegetable oil-based UV-curing resins at home and abroad, but some problems still exist. For example, 95% of the soybean oil produced is used as edible oil, and only 5% is used for other applications. As the most valuable edible oil resource, the remaining 5% of soybean oil may not be used for research purposes in the future. In addition, the overall cost of vegetable oil-based UV-curable materials is high, which further limits development. However, with the continuous progress of science and technology, more excellent solutions will be developed, and vegetable oil resources instead of oil resources are a major development trend in the future. For example, vegetable oil-based epoxy resins can be used as an alternative to commercial epoxy resins to effectively reduce the use of harmful chemicals such as epichlorohydrin and bisphenol A. It can also be used to synthesize composite materials, combined with reinforced fillers (such as graphene oxide, carbon nanotubes, butadiene rubber, etc.) and bio-based fillers (such as cellulose fibers, hemp fibers, lignin and expanded starch) to form products with better performance, making them more competitive in lamination, electronics, biomedical, antiseptic, hydrogel, shape memory polymers and aerospace.

## Figures and Tables

**Table 1:** Selection and modification of vegetable oil raw materials.

The Specific Research Direction:	Selection and Modification
Diversification of raw materials	study the influence of structural properties (unsaturation, hydroxyl value, epoxy value) of different vegetable oils (soybean oil, flaxseed oil, castor oil, tung oil, etc.) on the properties of the final resin [3,8,14].
Efficient Modification Technology	Epoxidation: Epoxy groups are introduced to improve reactivity. Acrylication/Methacrylate: Introduction of double bonds ((meth)acrylate groups required for light-curing) [2,6,16]. Optimize reaction conditions: (temperature, catalyst, polymerization inhibitor) to improve conversion and reduce side reactions. Hydroxylation: The hydroxyl group of the vegetable oil itself or the introduction of more hydroxyl groups is used to synthesize polyurethane acrylates [4,7]. Exchange/alcohololysis: Changing the fatty acid chain length or introducing polyols. Development of highly functional monomers: design and synthesis of vegetable oil derivatives with higher double bond functionality to increase curing speed and crosslinking density, and improve hardness, heat resistance and chemical resistance.

**Table 2:** Summary of mechanical properties of epoxidized linseed oil (ELO) crosslinked with a mixture of methyl nadic anhydride (MNA) and maleinized linseed oil (MLO).

MNA/MLO [wt/wt]	Flexural Strength [MPa]	Flexural Modulus [MPa]	Shore D
50/0	60.8 (5.8)	1772.0 (132.0)	82.2 (2.5)
45/5	54.9 (5.4)	1460.0 (73.5)	81.9 (1.3)
40/10	47.2 (3.1)	1027.0 (46.5)	78.2 (1.9)
35/15	31.9 (4.8)	718.2 (45.2)	76.1 (1.5)
30/20	23.8 (0.5)	485.8 (33.0)	71.4 (1.8)
25/25	14.5 (0.6)	272.3 (20.9)	64.1 (1.5)

**Table 3:** Modification methods and applications of different vegetable oils.

Type of Vegetable Oil	Structural Features	Modification Method	Superior Performance	Limitations	Fields of Application
Soybean oil, such as [24,27,34,40,67,68,80].	1. Unconjugated double bonds 2. Iodine value 120–140 (Semi-dry oil)	Epoxidation, acrylication, maleoylation, copolymerization with citric acid	High flexibility, low viscosity, bio-based content > 50%	Low functionality and poor chemical resistance	Wood coatings, water-based coatings, 3D printing, adhesives
Castor oil, such as [5,9,41,42,45,74].	1. Contains hydroxyl (ricinoleic acid) 2. Iodine value 80–90 (Non-drying oil)	Hydroxyl group is directly acrylated, and PU prepolymer is synthesized with isocyanate	High reactivity, fast curing speed (≤30 s) and high biodegradation rate	The distribution of hydroxyl groups is uneven, and the reaction conditions are difficult to control	Biodegradable adhesives, coatings
Tung oil, such as [53,54,61,75].	1. Conjugated trienes 2. Iodine value 160–175 (Dry oil)	Diels–Alder reaction, thiolene click reactions, acrylication	High functionality, high hardness, adhesion grade 0	The color is dark, the synthesis steps are complex	High hardness coatings, electronic packaging inks
Linseed oil, such as [55,56,58,59,60].	1. Highly unsaturated unconjugated double bond (linolenic acid) 2. Iodine value 170–190 (Dry oil)	Epoxidation, acrylication, compound with nano-SiO_2_	High cross-linking density, good thermal stability (>180 °C)	High viscosity, need to add diluent	Industrial coatings, packaging inks
Cottonseed oil, such as [57,62].	1. Unconjugated double bonds 2. The composition of fatty acids varies greatly	Epoxidation, acrylication	Flexibility is superior to petroleum-based materials and is suitable for flexible substrates	Research is not yet complete	Coating of flexible printed electronic and paper products

## Data Availability

The original contributions presented in the study are included in the article, further inquiries can be directed to the corresponding author.
